# Dysplastic hips with a subchondral cyst demonstrate poorer functional scores and a trend toward increased benefit from periacetabular osteotomy

**DOI:** 10.1016/j.jcot.2026.103596

**Published:** 2026-08-12

**Authors:** Chiara Heller, Quentin Karisch, Justus Stamp, Thair Afifi, Marco Haertlé, Sufian S. Ahmad

**Affiliations:** a Department of Orthopaedic Surgery, Hannover Medical School, DIAKOVERE Annastift, Anna-von-Borries Str. 1-7, 30625, Hannover, Germany; b Department of Orthopaedic Surgery, Helios Hanseklinik Stralsund, Große Parower Strasse 47-53, 18435, Stralsund, Germany; c AVIATOR Advanced Clinician Scientist Program, Dean's Office for Academic Career Development, Hannover Medical School, Carl-Neuberg-Str. 1, 30625, Hannover, Germany; d Department of Orthopaedics and Trauma Surgery, St. Josef Hospital, Heidbergweg 22-24, 45257, Essen, Germany

**Keywords:** Periacetabular osteotomy, Hip dysplasia, Hip preservation, Subchondral cysts

## Abstract

**Background:**

Subchondral cysts of the femoral head have been commonly regarded as indicators of joint degeneration in developmental dysplasia of the hip (DDH), attributed to excessive stress and mechanical overload. Whether such cysts constitute a contraindication to periacetabular osteotomy (PAO) in young hips remains controversial. The aim of this study was to determine early outcomes after PAO in young dysplastic hips in which a subchondral femoral head cyst with an intact cartilage cap was identified.

**Methods:**

An institutional PAO database was utilized, and hips undergoing PAO between January 2022 and December 2023 were identified. Preoperative magnetic resonance imaging (MRI) studies were reviewed for the presence of subchondral femoral cysts, and hips were stratified into groups with and without such cysts. Radiographic and demographic data were compared between groups. Preoperative and 1-year postoperative patient-reported outcome measures (PROMs; mHHS, iHOT-12) were collected and compared. Proportions of hips achieving the minimal clinically important difference (MCID) and patient acceptable symptom state (PASS) following PAO were calculated and compared.

**Results:**

A total of 107 hips were included; 21 (20%) were found to have subchondral femoral cysts and 86 (80%) were found not to have cysts. No significant differences in demographic or radiographic parameters were identified between groups. PROMs for hips with subchondral cysts were observed to be slightly lower pre- and postoperatively. Significant improvements in PROMs were observed in both groups at 1-year follow-up. A higher proportion of hips with subchondral cysts achieved MCID for both mHHS and iHOT-12. For PASS, the proportion of hips reaching the threshold was higher in the cyst group for mHHS, whereas it was higher in the non-cyst group for the iHOT-12.

**Conclusion:**

Periacetabular osteotomy yielded significant improvements in PROMs despite the presence of a structural cystic subchondral lesion under an intact cartilage cap. The greater improvements in mHHS and iHOT-12 observed among hips with subchondral cysts are suggested to reflect a higher baseline symptom burden, indicating that greater potential for functional improvement may be achieved once dysplastic biomechanics and associated overload and instability are corrected.

## 1 Introduction

Developmental dysplasia of the hip (DDH) is a well-established pathomorphology associated with symptomatic hip instability and the premature onset of osteoarthritis. Re-orientation osteotomy of the acetabulum aim to expand the functional weight-bearing surface area by optimizing the position of the lunate surface. This redistribution of mechanical forces enhances joint congruity, which ultimately contributes to improved joint stability 1, 2, 3.

Periacetabular osteotomy (PAO) represents the gold-standard surgical intervention for acetabular reorientation, as it permits significant correction while preserving the integrity of the posterior column.^1^^,^^4^ The clinical efficacy and longitudinal outcomes of PAO have been extensively reported.

Generally, the success of the procedure is inversely correlated with the severity of preoperative degenerative changes, which have traditionally been staged using the Tönnis classification on conventional radiographs^5^, ^6^, ^7^

The objective of this study was to evaluate the short-term outcomes of PAO in patients with subchondral femoral cysts identified via magnetic resonance imaging (MRI). It was hypothesized that the presence of an MRI-confirmed subchondral femoral cyst is associated with significantly lower patient-reported outcome measures (PROMs) and Minimum Clinically Important Differences (MCIDs) are higher in hips with subchondral cysts indicating greater potential for improvement.

## 2 Methods

The study was a retrospective cohort study based on a prospectively maintained registry conducted after approval of the institutional ethics committee. The protocol was approved prior to conduction of the study (12516-BO-K-2026).

Data was retrieved from the institutional longitudinal hip registry for hips treated by a single surgeon between 01/2022 and 12/2023. All hips were undergoing rectus sparing PAO via modified Smith-Petersen approach.^1^ In only one case, additional subchondral filling of the cyst was required. Demographic and radiographic data were prospectively included via this institutional registry, including body mass index (BMI), age and gender.

**Fig. 1:**
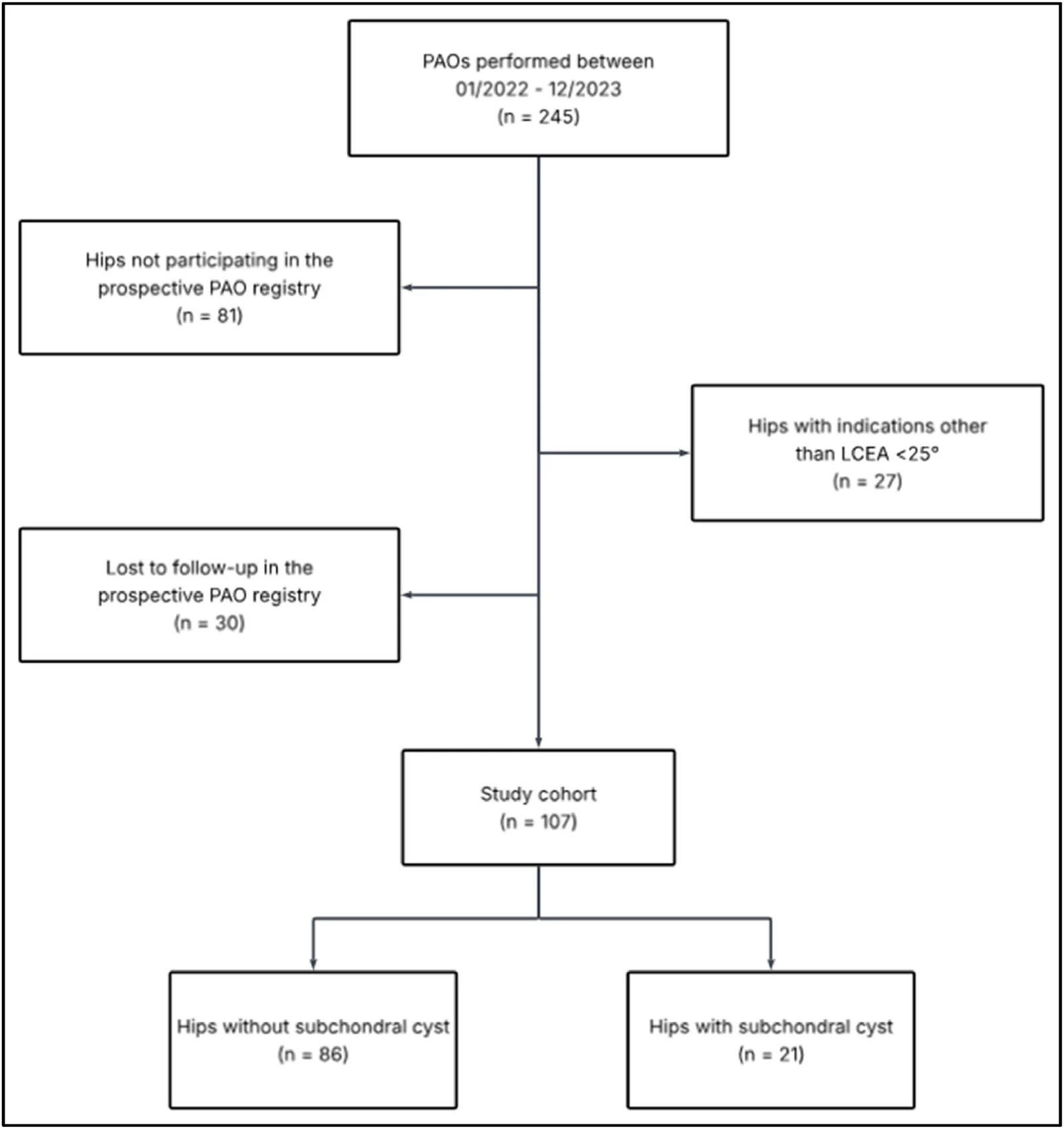
Flowchart of hips undergoing PAO matching the inclusion criteria. LCEA, lateral center-edge angle.

All patients with a minimum follow-up of one year were reviewed, provided that they had given written consent for data use. This study addressed dysplastic hips in adolescents and adults with a lateral center–edge angle (LCEA) < 25°. Hips were included if they displayed the presence of a subchondral cystic lesion at the time of PAO surgery. Only hips with preoperative MRI were considered for this study. Although MRIs were not standard for diagnosis, pre-operative MRIs were performed on most patients in order to gain a better understanding of the three-dimensional anatomy of the hip and to assist in planning the operation. Exclusion criteria included inadequate follow-up and the concomitant procedures that may alter comparability with a standard cohort (i.e surgical hip dislocation, correction of femoral antetorsion, relative neck lengthening), paralabral cysts were not considered subchondral cysts and were excluded from the study cohort, the same for acetabular cysts to ensure homogeneity. Hips with no subchondral femoral cyst undergoing PAO, were included for comparison, provided that the hips also underwent isolated PAO surgery (Fig. 1).

**Fig. 2:**
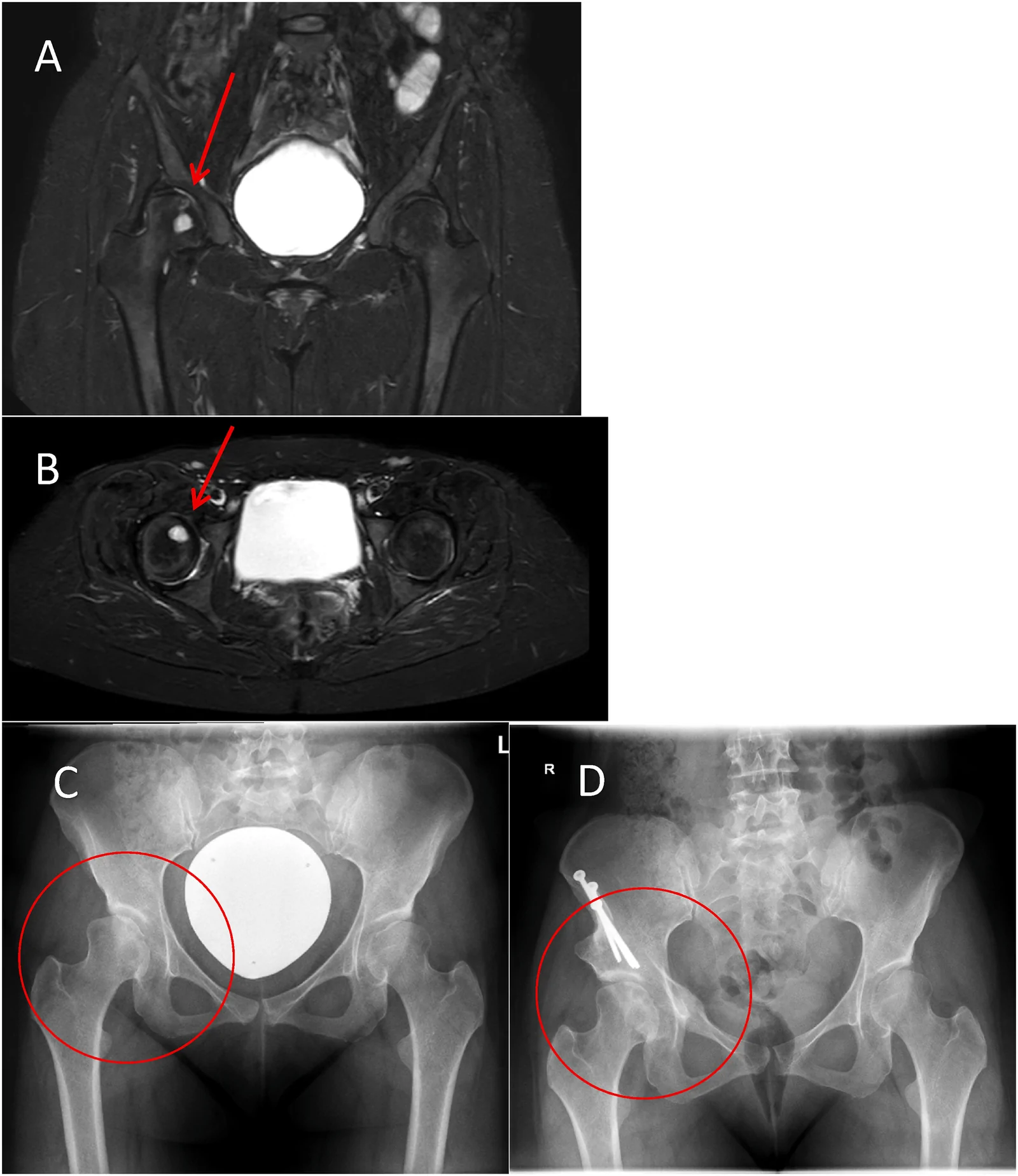
Example of subchondral cyst in the preoperative MRI A) preoperative MRI, coronar plane B) preoperative MRI, transversal plane C) preoperative x-ray, a.p. pelvis D) 1-year postoperative x-ray, a.p. pelvis.

The presence of a subchondral femoral cyst was defined as a fluid-filled cavity in the bony region of the femoral head on magnetic resonance imaging (MRI) (Fig. 2). Two investigators noted the presence of a cyst based on a binary scale. All patients with a cyst undergoing PAO had no further signs of degeneration and an intact cartilage cap over the cyst with an overall healthy chondral layer and no osteophytes. A defect in the chondral layer or cartilage was considered a PAO contraindication regardless of size since it indicates more advanced intra-articular damage and may lower the expected benefit of PAO.^7^ To this point, patient-reported outcomes for hips with subchondral cysts have not been investigated yet. This would be beneficial for understanding potential or limitations for hip preserving surgery for joints with cartilage damage.

Radiographic measures were tabulated, including the lateral center edge angle (LCEA), acetabular index (AI), extrusion index (EI), anterior wall index (AWI) and posterior wall index (PWI). The following patient-reported outcome measures (PROMs) were retrieved preoperatively and at one-year follow-up: the University of California, Los Angeles Activity Scale (UCLA), the Hip Disability and Osteoarthritis Outcome Score – Physical Function Shortform (HOOS-PS), the Western Ontario and McMaster Universities Osteoarthritis Index (WOMAC), the International Hip Outcome Tool 12-item (iHOT-12), the Harris Hip Score (HHS), the Modified Harris Hip Score (mHHS) and the Postel–Merle d'Aubigné (PMA) score.

The minimal clinically important difference (MCID) and patient acceptable symptom state (PASS) cut-offs for PAO surgery have previously been described for both the mHHS and iHOT-12 scores. An MCID of 8 and a PASS of 44 for the mHHS were applied.^8^^,^^9^ For the iHOT-12, an MCID of 13 and a PASS of 44 points were applied.^10^^,^^11^

Several sources of bias must be considered when interpreting the results of this study. As this study is a retrospective cohort study, selection bias should be considered. Moreover, confounding factors may have influenced the observed outcomes. Unmeasured variables like level of activity or biomechanical factors have not been considered in the present study. The relatively small sample size may increase risk for type II error potentially masking small but clinically relevant differences between groups.

### 2.1 Statistical analysis

Results were tabulated and presented as means with standard deviation (Table 1). Preoperative and 1-year postoperative PROMs were compared as medians with interquartile range (Table 2). Changes in PROMs after PAO were calculated and shown as box plots. For all analyses Mann-Whitney-U-test was performed.

**Table 1:** Demographic data of hips undergoing PAO without subchondral cysts vs. with cysts.

	*without cysts*		*with cysts*		p-value
	n	mean (±SD)	n	mean (±SD)	
sex					
female	71 (66%)		14 (13%)		
male	15 (14%)		7 (7%)		**0.197

demographic data					
BMI (kg/m^2^)		24.56 (±5.56)		23.84 (±4.53)	*0.259
age		28.38 (±7.74)		30.90 (±7.23)	*0.176

preoperative radiographic parameters					
LCEA (°)		18.28 (±5.68)		18.89 (±5.97)	*0.551
AI (°)		13.16 (±5.44)		14.41 (±7.37)	*0.599
EI (%)		27.75 (±5.97)		25.87 (±6.70)	*0.149
AWI (%)		39.66 (±12.67)		43.81 (±15.20)	*0.132
PWI (%)		84.55 (±16.80)		84.96 (±14.86)	*0.925
postoperative radiographic parameters					
LCEA (°)		33.06 (±6.49)		34.10 (±7.36)	*0.531
AI (°)		1.44 (±5.75)		2.58 (±6.51)	*0.412
EI (%)		13.23 (±5.70)		13.25 (±6.86)	*0.793
AWI (%)		38.26 (±13.83)		36.60 (±13.25)	*0.798
PWI (%)		83.26 (±22.07)		86.96 (±20.14)	*0.628

Note: * Mann-Whitney *U* test, ** chi-square test.

LCEA, lateral center-edge angle; AI, acetabular index; EI, extrusion index; AWI, anterior wall index; PWI, posterior wall index; BMI, body mass index; UCLA, University of California and Los Angeles activity-level rating score; HOOS‐PS, hip disability and osteoarthritis outcome score‐physical function shortform; WOMAC, Western Ontario and McMaster Universities Osteoarthritis Index; HHS, Harris hip score; iHOT‐12, International Hip Outcome Tool 12; mHHS, modified Harris hip score; PMA, Merle d'Aubigné and postel score.

**Table 2:** Comparison of radiographic parameters and PROMs preoperative and postoperative for hips undergoing PAO with subchondral cysts and without subchondral cysts.

	*preoperative*		*p-value*	*postoperative*		*p-value*
	*without cysts*	*with cysts*		*without cysts*	*with cysts*	
	*median (Q1, Q3)*	*median (Q1, Q3)*		*median (Q1, Q3)*	*median (Q1, Q3)*	
PROMs						
UCLA	6.0 (5.0, 7.0)	6.0 (4.25, 7.75)	*0.750	7.0 (6.5, 7.5)	6.0 (4.5, 7.5)	*0.015
HOOS-PS	60.0 (44.35, 75.65)	57.5 (46.25, 68.75)	*0.630	80.0 (72.0, 88.0)	85.0 (67.0, 103.0)	*0.974
WOMAC	16.7 (1.2, 32.3)	26.9 (10.65, 43.15)	*0.134	9.6 (1.0, 18.2)	12.5 (3.45, 21.55)	*0.167
iHOT-12	46.8 (28.55, 65.05)	34.6 (19.9, 49.3)	*0.056	71.0 (49.75, 92.25)	67.5 (39.65, 95.35)	*0.572
HHS	71.0 (63.85, 77.15)	68.0 (61.75, 74.25)	*0.536	86.0 (75.0, 97.0)	86.0 (74.5, 97.5)	*0.863
mHHS	71.0 (63.0, 79.0)	67.0 (57.1, 76.9)	*0.446	84.0 (77.5, 90.5)	85.5 (69.0, 92.0)	*0.293
PMA	15.0 (14.5, 15.5)	15.0 (14.5, 15.5)	*0.591	17.0 (16.0, 18.0)	17.0 (16.0, 18.0)	*0.803

Note: * Mann-Whitney *U* test.

UCLA, University of California and Los Angeles activity-level rating score; HOOS‐PS, hip disability and osteoarthritis outcome score‐physical function shortform; WOMAC, Western Ontario and McMaster Universities Osteoarthritis Index; HHS, Harris hip score; iHOT‐12, International Hip Outcome Tool 12; mHHS, modified Harris hip score; PMA, Merle d'Aubigné and postel score.

Proportions of hips achieving MCID and PASS for mHHS and iHOT-12 were calculated and stratified by the presence of subchondral cysts. Differences between groups were analyzed using the Fisher's exact test.

All statistical analyses were performed using IBM SPSS statistics. Graphs were designed using GraphPad Prism.

## 3 Results

There were 21 (20%) hips with a subchondral cyst in the femoral head and 86 (80%) without (Fig. 1). Demographic data are presented in Table 1. Of all hips included, 85 (79%) were female and 22 (21%) were male. The mean age was 31 years for hips with subchondral femoral cysts and 28 years for hips without cysts (p = 0.176). BMI was 23.84 kg/m2 for hips with cysts and 24.56 kg/m2 for hips without cysts, with no significant difference observed (p = 0.259).

No relevant differences were observed between hips with and without subchondral cysts for any radiographic measure of coverage (LCEA, EI, AI, AWI, PWI) (Table 1). Hips without subchondral cysts demonstrated less pronounced changes in PROMs than hips with cysts (Table 2).

**Fig. 3:**
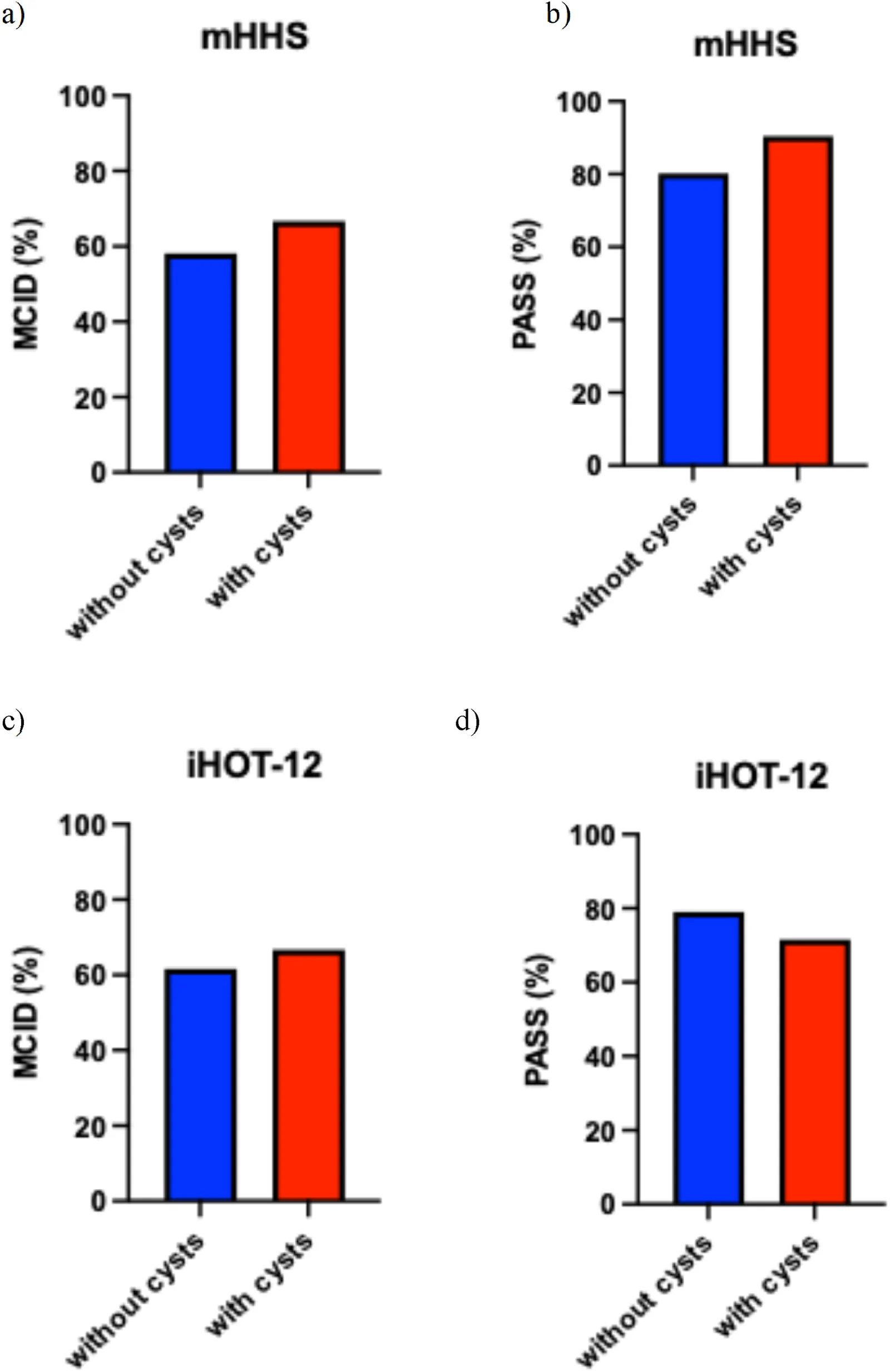
Proportion of hips achieving MCID and PASS after PAO for mHHS and iHOT-12 stratified as hips without and with subchondral cysts. *Fishers exact test: a) p = 0.354, b) p = 0.621, c) p = 0.560, d) p = 0.803.

Postoperative UCLA scores were significantly lower in hips with subchondral cysts (p = 0.015), whereas no significant differences were observed for the other PROMs (p > 0.05). A higher percentage of hips with cysts achieved the MCID (Fig. 3).

For the mHHS, achievement of the MCID (≥8 points) was observed in 58.14% of hips without cysts and 66.67% of hips with cysts. Achievement of the PASS (>44 points) was observed in 80.23% of hips without cysts and 90.48% of hips with cysts.

For the iHOT-12, 61.63% of hips without subchondral cysts and 66.67% of hips with subchondral cysts achieved an MCID (>13 points). The PASS (≥44 points) was reached by 79.07% of hips without subchondral cysts and 71.43% of hips with subchondral cysts.

**Fig. 4:**
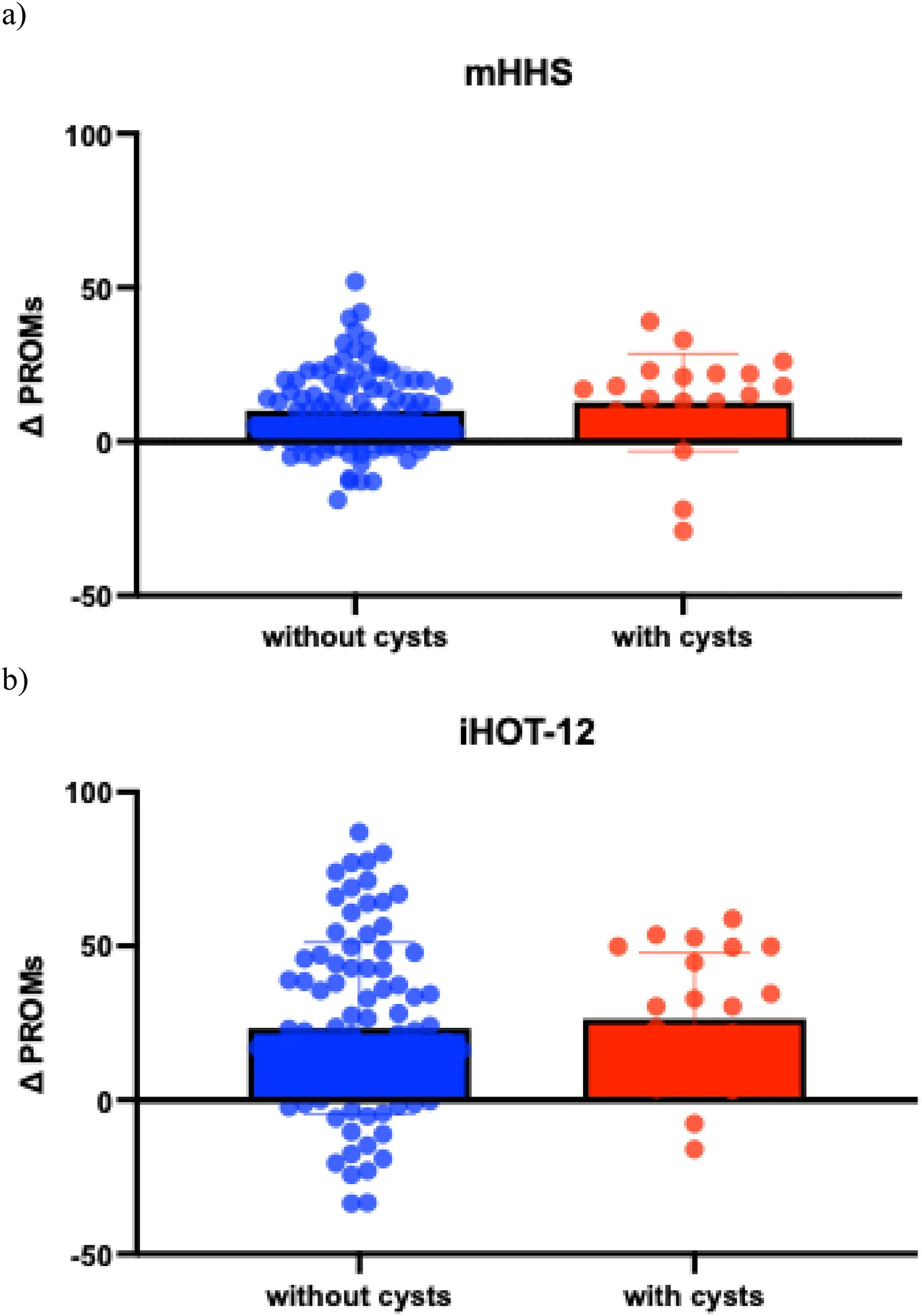
Change in PROMs after PAO in hips with and without subchondral cysts. *Mann-Whitney *U* test: a) 0.1622, b) 0.5022.

On detailed analysis of PROM changes after PAO, both hips with and without cysts demonstrated clear overall improvement (Fig. 4). A slightly greater mean improvement in mHHS was observed for hips with subchondral cysts. Similarly, larger increases in iHOT-12 scores were observed for hips with cysts, while hips without cysts exhibited a broader range of individual responses. Clinically relevant improvements in PROMs were apparent in both groups.

## 4 Discussion

The most important finding of this study was that PAO leads to improvement in PROMs, even in the presence of a structural subchondral lesion. The slightly greater improvements in mHHS and iHOT-12 among hips with subchondral cysts suggests a higher baseline symptom burden in these patients. Consequently, they may have greater potential for functional improvements once the dysplastic biomechanics and associated overload and instability are improved.

Subchondral cysts have been described in a variety of studies as radiographic signs of joint degeneration.^12^^,^^13^ However, the literature does not rule out potential benefit from joint preserving surgery in these hips, with otherwise a good cartilage cap and no further degeneration.^14^ Hartigan et al. (2017) emphasized a more cautious interpretation of subchondral cysts in MRI, reporting higher rates of conversion to hip arthroplasty in hips with subchondral cysts than in hips without cysts. However, significant improvement in symptoms was still demonstrated by hips that did not require subsequent revision.^15^

Higher rates of MCID achievement and greater improvements in PROMs were observed in hips with subchondral cysts, reflecting meaningful symptomatic relief. Clinically, the presence of subchondral cysts on MRI should not be regarded as an absolute contraindication to hip-preservation procedures such as PAO in young hips that do not demonstrate additional degenerative change with an intact cartilage cap. Instead, the findings underscore the importance of careful patient selection.

In subgroup analysis comparing hips with and without subchondral cysts, no significant differences were found in radiographic measures regarding the degree-, phenotype of dysplasia, or patient demographics. Thus, the presence of cystic lesions cannot be explained by two-dimensional conventional radiographic measurements alone. Further research is warranted to investigate additional factors not captured by standard imaging, such as focal mechanical overload within the weight-bearing zone, three-dimensional joint incongruity and soft-tissue factors.

The principal strength of this study is the well-defined cohort in which hips with and without subchondral cysts were compared. Internal validity is enhanced by the performance of all procedures by a single surgeon using a consistent surgical technique and standardized pre- and postoperative evaluations. Clinically meaningful assessment of patient outcomes was enabled through the use of validated PROMs (mHHS and iHOT-12). The topic and its limitations are thereby contributed to the literature on a clinically relevant and currently underexplored aspect of hip preservation.

Despite these strengths, several limitations must be acknowledged. Selection and information bias may be introduced by the retrospective design. The sample size—particularly within the subgroup of hips with subchondral cysts—may be insufficient for detection of small intergroup differences. Since the subgroup of hips with subchondral cysts was small, the statistical robustness of descriptive analysis was limited. Radiographic data were also not normally distributed. However, mean and standard deviation were reported to facilitate comparability. These results should therefore be interpreted as descriptive rather than definitive. Additionally, only short-term outcomes were examined; evaluation of long-term results, including progression to osteoarthritis, will require extended follow-up periods and collection of long-term PROMs. Given the limited sample size of hips with subchondral cysts, a detailed analysis on size, location and distribution of the cyst would have provided limited insights. Therefore, the presence of subchondral cysts was recorded as a binary radiographic finding only. This limitation should be considered when interpreting the role of cysts in our study.

## 5 Conclusion

In conclusion, clinically important differences in PROMs following PAO in dysplastic hips were observed in both groups. Although subchondral cysts are indicative of advanced mechanical overload, adequate correction of acetabular coverage was associated with achievement of the minimal clinically important difference in two-thirds of affected hips. These findings suggest that the presence of subchondral cysts should not be considered an absolute contraindication to PAO in young patients provided no further degeneration is present and the cartilage cap is intact. Nevertheless, further research is required to define the limits of this indication, and mid- and long-term outcomes remain to be determined.

## Patient's consent

Written informed consent was obtained from all individual participants included in the study. All participants were informed about the purpose of the study and the confidential nature of the data collection.

## Ethical statement

The authors declare that the study was conducted in accordance with the ethical standards of the institutional research committee and with the Helsinki Declaration. Ethical approval was obtained from the ethics committee ((I0405_BO_K_2022). All procedures performed in this study were carried out following the ethical principles of clinical research. Informed consent was obtained from all individual participants included. The authors confirm that patient data were anonymized to ensure confidentiality and that no identifying information is included in the manuscript.

## CRediT author statement

**Conceptualization:** Sufian S. Ahmad, Chiara Heller.

**Data Curation:** Chiara Heller, Quentin Karisch, Justus Stamp, Marco Haertlé.

**Formal Analysis:** Sufian S. Ahmad, Chiara Heller, Quentin Karisch, Justus Stamp, Marco Haertlé, Thair Afifi.

Funding **Acquisition:** Sufian S. Ahmad, Marco Haertlé.

**Investigation:** Chiara Heller.

**Methology**: Sufian S. Ahmad, Chiara Heller.

**Project Administration:** Sufian S. Ahmad, Chiara Heller.

**Resources:** Sufian S. Ahmad, Chiara Heller.

**Software:** IBM SPSS, GraphPad PRISM.

**Supervision:** Marco Haertlé.

**Validation:** Quentin Karisch, Justus Stamp, Thair Afifi.

**Writing - Original Draft:** Sufian S. Ahmad, Chiara Heller.

**Writing - Review & Editing:** Marco Haertlé, Justus Stamp, Quentin Karisch, Thair Afifi.

## Funding

No funding was received for the conduction of the study.

## Declaration of competing interest

Prof. Sufian Ahmad reports consulting and development relationships with Medacta. The remaining authors declare no competing interests.
